# Electroacupuncture Regulates Cannabinoid Receptor 1 Expression in a Mouse Fibromyalgia Model: Pharmacological and Chemogenetic Modulation

**DOI:** 10.3390/life14111499

**Published:** 2024-11-17

**Authors:** Yu-An Yeh, Hsin-Cheng Hsu, Ming-Chia Lin, Tzu-Shan Chen, Wei-Cheng Lin, Hsiang-Ming Huang, Yi-Wen Lin

**Affiliations:** 1 Graduate Institute of Acupuncture Science, College of Chinese Medicine, China Medical University, Taichung 404328, Taiwan; 0002762@tool.caaumed.org.tw; 2 School of Post-Baccalaureate Chinese Medicine, College of Chinese Medicine, China Medical University, Taichung 404328, Taiwan; hch@mail.cmu.edu.tw; 3 Department of Traditional Chinese Medicine, China Medical University Hsinchu Hospital, China Medical University, Hsinchu 302056, Taiwan; 4 Department of Nuclear Medicine, E-DA Hospital, College of Medicine, I-Shou University, Kaohsiung 82445, Taiwan; ed101186@edah.org.tw; 5 Department of Medical Research, E-Da Hospital, I-Shou University, Kaohsiung 82445, Taiwan; 6 Graduate Institute of Sports and Health Management, National Chung Hsing University, Taichung 402202, Taiwan; wilson890704@gmail.com; 7 Department of Neurosurgery, China Medical University Hsinchu Hospital, China Medical University, Hsinchu 302056, Taiwan; 8 Chinese Medicine Research Center, China Medical University, Taichung 404328, Taiwan

**Keywords:** electroacupuncture, fibromyalgia, cannabinoid receptor 1, thalamus, somatosensory cortex, anterior cingulate cortex

## Abstract

Fibromyalgia is a chronic illness usually accompanied by long-lasting, general pain throughout the body, often accompanied by anxiety, depression, fatigue, and sleep disruption. Meanwhile, doctors and scientists have not entirely discovered detailed mechanisms; patients always have an exaggerated sensation to pervasive pain without satisfied medical service. Given the lack of knowledge on its underlying mechanism, current treatments aim to provide pain and/or symptom relief. The present study aimed to clarify the role of cannabinoid receptor 1 (CB1) signaling in a mouse fibromyalgia pain model. To develop the mouse fibromyalgia model, mice were subjected to intermittent cold stress (ICS). Our results indicated that mechanical (2.09 ± 0.09 g) and thermal hyperalgesia (4.77 ± 0.29 s), which were evaluated by von Frey and Hargraves’ tests, were induced by ICS, suggesting successful modeling. The hurting replies were then provoked by electroacupuncture (EA) but not for sham EA mice. Further, in a Western blot analysis, we found significantly decreased CB1 protein levels in the thalamus, somatosensory cortex, and anterior cingulate cortex. In addition, the levels of pain-related protein kinases and transcription factor were increased. Treatment with EA reliably increased CB1 expression in various brain regions sequentially alleviated by nociceptive mediators. Furthermore, the administration of a CB1 agonist significantly attenuated fibromyalgia pain, reversed EA analgesia by the CB1 antagonist, and further reversed the chemogenetic inhibition of SSC. Our innovative findings evidence the role of CB1 signaling in the interaction of EA and fibromyalgia, suggesting its potential for clinical trials and as a treatment target.

## 1. Introduction

Pain is an unpleasant sensory and emotional experience produced by trauma, injury, or sickness. Pain may result from tissue damage, local inflammation, muscle injury, or infection. Pain is a survival mechanism acting as an alarm signal, aiming to prevent further damage. Nociceptive terminals include elements that can reliably detect noxious stimuli, which can activate nociceptors and deliver painful signals as a nerve impulse through the ascending pain pathway via the spinal cord, and brainstem, and finally to the brain [1,2]. Several regions are involved in analgesia, including the somatosensory cortex (SSC) and anterior cingulate cortex (ACC), and treatment with pregabalin significantly attenuated the activation of SSC, the thalamus, and ACC [3]. In addition, IL-1β potentiation has been detected in the thalamus of rats with induced neuropathic pain, and lidocaine was abridged in the thalamus of chronic constriction injury (CCI) mice, suggesting a crucial role of the thalamus in pain sensation [4]. Further, increased microglial activity was found in streptozotocin-injected mice, which show mechanical and thermal hyperalgesia. Later, microglia activation increases the secretion of inflammatory cytokines in the thalamus [5]. The augmentation of nociceptive receptors such as sodium and calcium channels, transient receptor potential, and glutamate receptors along with a reduction in inhibitory cannabinoid receptors (CBs) responding to neuroplasticity of the malfunction of the somatosensory cortex (SSC) lead to neuropathic pain. These nociceptive signals are further sent to the higher-pain-perception anterior cingulate cortex (ACC) region [6].

Fibromyalgia (FM) is a long-term, chronic, widespread musculoskeletal pain condition characterized by symptoms such as pain in peripheral muscles, tenderness, anxiety, depression, fatigue, and sleep disorders. Much is unknown about the condition, which explains the lacking precise protocols to confirm a diagnosis. FM affects 2–8% of the population; most patients are middle-aged females [7,8]. Currently, the widespread pain index (WPI) and symptom severity scale (SS) are used to diagnose FM severity. The WPI considers 19 pain points while SS focuses on the degree of fatigue, walking, and cognitive symptoms. FM is diagnosed as WPI ≥ 7 and SS ≥ 5 or WPI 3–6 and SS ≥ 9 in the previous 3 months. Some medications, meditation, exercise, therapy, and lifestyle changes can improve symptoms and increase quality of life [9,10,11]. Fibromyalgia may result from a combination of factors, including heredity, inflammation, and psychological stress. Antidepressants including duloxetine (Cymbalta) and milnacipran (Savella) are utilized to relieve FM symptoms by rebalancing neurotransmitters/neuromodulators [12,13]. Gabapentinoids, sedatives, selective serotonin reuptake inhibitors, serotonin norepinephrine reuptake inhibitors, and tricyclic compounds are also applied for FM, although they have several side effects [14,15].

Cannabinoid receptors (CBs) can be activated at both peripheral and central sites from cannabis plants. CBs are G-protein-coupled receptors with seven transmembrane domains that can be subgrouped into CB1 and CB2 receptors, encoded by *CNR1* and *CNR2*. CB1 was first detected in the brain in regions like the hippocampus, cerebellum, hypothalamus, amygdala, periaqueductal gray (PAG), and cerebral cortex [16,17], followed by the discovery of peripheral CB2 receptors. CBs can be activated by their endogenous ligands, arachidonylethanolamide (AEA) and 2-arachidonoylglycerol. Endocannabinoids bind at CB1 in CNS neurons or microglia and CB2 in peripheral immune cells. Endocannabinodals can activate CB1 receptors to attenuate adenylate cyclase, cAMP, and potassium and voltage-gated calcium channels to inhibit synaptic transmission. CBs have been involved in the regulation of pain, mood, appetite, and immune responses in several pathophysiological conditions [18,19]. Endocannabinoids are expressed in the nociceptive pathways in the primary afferent of the dorsal horn of the spinal cord, as well as in supraspinal areas involved in pain perception and modulation. Recent research indicates that endocannabinoids can produce short-term analgesic effects via presynaptic CB1 receptors [20,21].

Acupuncture was recommended by the World Health Organization and it consists of inserting a thin steel needle into specific local points on the body to alleviate pain. Acupoints are precise areas that stimulate connective tissue, muscle, and peripheral nerves for qi flow and therapeutic purposes. Electroacupuncture (EA) was developed to standardize parameters to reduce inflammatory pain, neuropathic pain, and FM pain in mouse models. Recently, EA through the optogenetic stimulation of nerve terminals at the ST36 acupoint was reported to trigger the vagal–adrenal axis in mice [22]. Further, another study reported an anti-inflammatory effect of EA for controlling systemic inflammation in a mouse sepsis model [23]. Accordingly, we indicated that EA could be useful in a mouse inflammatory pain model through adenosine and opioid receptors [24,25]. We recently showed that EA could increase the release of adenosine triphosphate, IL-1β, IL-6, glutamate, substance P, and histamine in the acupoint microenvironment [26]. In addition, EA reliably alleviated FM pain by reduction in inflammatory cytokines such as ILs, tumor necrosis factor-α (TNF-α), and interferon gamma (IFN-γ) in mouse plasma [27]. EA was reported to relieve several kinds of diseases with different mechanisms [28,29,30,31,32].

In this study, our hypothesis is that EA can relieve FM pain through the CB1-associated pathway. In addition, novel mechanisms behind pain-relieving properties of EA to treat FM pain should be solved. Thus, EA may reduce FM pain through CB1 receptors in the mouse ascending pathways. Here, we observed that pain behaviors were found and reduced the CB1 receptor in the mice ascending pathway including thalamus, SSC, and ACC areas. Decreased CB1 expression resulted in increased levels of pain-associated protein kinases and transcription factors. These effects were reversed by EA but not by sham treatment. The intracerebral injection of the CB1 agonist meaningfully diminished FM pain, reversed EA analgesia by CB1 antagonist AM251, and further attenuated the chemogenetic inhibition of SSC. Therefore, our results in a mouse FM pain model support the potential use of EA for FM.

## 2. Materials and Methods

### 2.1. Animals and FM Pain Induction

For experiments, we employed 8–12-week-old, female C57B/L6 mice (18–20 g) purchased from BioLasco (Yilan, Taiwan). Mice were kept in cages under a 12 h light and dark cycle at 25 °C and 60% humidity. Nine mice per group was considered the minimum needed for an α level of 0.05 and a power of 80%. All mice were legalized by the Institute of Animal Care and Use Committee of China Medical University (Permit no. CMUIACUC-2021-336), Taiwan, following the Guide for the Use of Laboratory Animals (National Academy Press). We did our best to decrease the number of mice used in our experiments and to cause minimal animal discomfort. The laboratory workers were blind to treatment allocation during the experiments and analysis. Mice were randomly assigned into four groups: (1) normal mice (normal), (2) cold stress-induced FM pain (FM), (3) cold stress-induced FM pain with 2 Hz EA (FM + 2 Hz EA), (4) cold stress-induced FM pain with sham EA (FM + sham EA). To develop the mouse FM model, mice were placed in a 4 °C refrigerator while normal mice continued at room temperature. At 10 a.m. the next day, FM mice were exposed to room temperature for 30 min and then moved back to 4 °C for 30 min. The procedure was repeated until 4 p.m. before they were transposed again overnight from 4 p.m.; the process was performed until the third day.

### 2.2. Electroacupuncture

Mice were anesthetized with 1% isoflurane gas via a head tube. Two 1-inch stainless steel needles (32G, Yu Kuang Chem. Ind. Corp., Taipei, Taiwan) were inserted directly into the mouse ST36 acupoint. The mice ST36 was located 3–4 mm under the patella, between the fibula and tibia bones, at the anterior side of the anterior tibial muscle at a depth of 3–4 mm. EA parameters were 1 mA in intensity, 2 Hz in frequency, and 100 μs in width of a persistent square pulse, applied continuously for 20 min with an electronic machine, a Trio 300 stimulator (Ito, Tokyo, Japan). To ensure a therapeutic effect, EA was applied for two days.

### 2.3. Pain Behavior Test

Mechanical and thermal hyperalgesia were examined at baseline, day 1, and day 2 after FM establishment and EA treatment. Mice were placed in Plexiglas boxes above a steel mesh in a quiet room at room temperature to keep them calm and to adjust to the new location. While the mice were not moving, scratching, sleeping, or grooming, electronic von Frey filament tests were finished twice per succession, at 10 min intervals (IITC Life Science Inc., Woodland Hills, CA, USA). The Hargreaves’ test was then used to measure thermal latency. Mice were divided into separate Plexiglass fields to limit interactions. The IITC Plantar Analgesia Meter (IITC Life, Sciences, SERIES8, Model 390G, Burbank, CA 91367, USA) counted the removal latency of the mice’s hind paw from radiant thermal light. Each protocol was repeated twice per mouse. The device was shut down at 20 s to avoid damaging mice’s paws.

### 2.4. Western Blot Analysis

The mouse thalamus, SSC, and ACC brain were dissected to extract tissue proteins on day 4. Samples were then placed on ice and later stored at −80 °C before protein extraction. All proteins were extracted in a cold ratio immunoprecipitation (RIPA) lysis buffer (50 mM Tris-HCl, pH 7.4; 1% NP-40; 250 mM NaCl; 5 mM Ethylenediaminetetraacetic acid (EDTA); 50 mM NaF; 1 mM Na_3_VO_4_; 0.02% NaN_3_; and 1 × protease inhibitor cocktail). The proteins then underwent 8% SDS-Tris glycine gel electrophoresis and were transferred to a polyvinylidene difluoride (PVDF) membrane, which was then incubated with 5% non-fat milk in a TBS-T buffer (10 mM Tris, pH 7.5; 100 mM NaCl; 0.1% Tween 20), and incubated with a primary antibody against anti-tubulin (1:5000, Merck, Rahway, NJ, USA), CB1 (1:1000, Alomone, Jerusalem, Israel), pPKA (1: 1000, Alomone, Jerusalem, Israel), pPI3K (1:1000, Millipore, Burlington, MA, USA), pPKC (1: 1000, Millipore, MA, USA), pAkt (1:1000, Millipore, MA, USA), pmTOR (1:500, Millipore, MA, USA), pERK (1:1000, Millipore, MA, USA), and pNFκB (1:1000, Millipore, MA, USA) in TBS-T with 1% bovine serum albumin (BSA) at 4 °C overnight. Next, membranes were incubated in a secondary peroxidase-conjugated anti-rabbit antibody, anti-mouse antibody, or anti-goat antibody (1:5000) for 2 h. Western blot signals were acquired with a chemiluminescent substrate kit (PIERCE) under LAS-3000 Fujifilm (Fuji Photo Film Co., Ltd. Tokyo, Japan). The bands’ concentrations of specific molecular weight were quantified with NIH Image J software version 1.53e (Bethesda, MD, USA). α-Tubulin was set as the internal controller. The considered percentages were counted by dividing from the same qualified group associated with normality [33,34].

### 2.5. Immunofluorescence

Mice were euthanized with isoflurane (5%) and intracardially perfused with 0.9% saline followed by 4% paraformaldehyde. The mouse brain was instantly dissected and fixed with 4% paraformaldehyde at 4 °C for three days. The fleshy tissues were next engaged in 30% sucrose for cryoprotection overnight at 4 °C. The brain was fixed in the optimal cutting temperature compound, quickly frozen via liquid nitrogen, and stored at −80 °C. Frozen brain samples were cut into 20 μm sections with a cryostat, then placed on glass slides. The samples were fixed with 4% paraformaldehyde and incubated with a blocking solution (3% BSA, 0.1% Triton X-100, and 0.02% sodium azide) for 1 h at room temperature. Afterward, the samples were incubated with the primary antibody against anti-CB1 (1:1000, Alomone, Jerusalem, Israel) and pERK (1:1000, Millipore, MA, USA) in 1% BSA overnight. The samples were further incubated with the secondary antibodies (1:500), 488-conjugated AffiniPure donkey anti-rabbit IgG (H + L), 594-conjugated AffiniPure donkey anti-goat IgG (H + L), and peroxidase-conjugated AffiniPure donkey anti-mouse IgG (H + L), for 2 h at room temperature and later fixed with cover slips for immunofluorescence visualization [33,34].

### 2.6. CB1 Receptor Agonist and Antagonist Administration

Adult C57BL/6 female mice (*n* = 6) were used for the CB1 agonist or antagonist test. After the induction of FM in mice, the CB1 agonist AEA (Sigma, St. Louis, MO, USA) was administered intracerebroventricularly (i.c.v.) at a concentration of 100 µM. Alternatively, the CB1 antagonist AM251 (Sigma, St. Louis, MO, USA; in 10 µL of saline) was immediately administered i.c.v. at a dose of 5 µg. AEA and AM251 were administered under light isoflurane anesthesia (1%).

### 2.7. Chemogenetic Operation

Experimental mice were anesthetized with 1% isoflurane for maintenance. Subsequently, their heads were immobile with a stereotaxic frame and a cannula implanted at the SSC site. The 23-G, 2 mm steel cannula was fixed 0.5 mm posteriorly and 1.5 mm laterally from the bregma at a depth of 175 μm below the cortical surface. The cannula was fixed to the skull with dental glue. The injection cannula was inserted and connected to a Hamilton syringe with a PE tube (PE10, Portex, Göteborg, Sweden). A total of 0.3 μL of the viral solution hM4Di DREADD (designer receptors exclusively activated by designer drugs: AAV8-hSyn-hM4D(Gi)-mCherry; Addgene Plasmid #50475, Watertown, MA, USA) was injected over a period of 3 min using a syringe pump (KD Scientific, Holliston, MA, USA). After injection, the injection cannula remained in the SSC for an additional 2 min to allow the viral solution to be fully injected. Clozapine N-oxide (CNO; Sigma C0832, Burlington, MA, USA) was used to activate the excitatory DREADD. CNO was thawed in 5% dimethyl sulfoxide (DMSO; Sigma D2650, Burlington, MA, USA) and later diluted with normal saline.

### 2.8. Statistical Analysis

The statistical analysis was performed using SPSS software 21.0. All results are presented as the mean ± standard error (SEM). Differences among groups were compared using an ANOVA test, followed by a post hoc Tukey’s test. *p* < 0.05 was considered the threshold for statistical significance.

## 3. Results

### 3.1. Electroacupuncture Alleviated Intermittent Cold Stress-Induced FM Pain in Mice as Shown in the Von Frey and Hargraves’ Tests

**Figure 1:**
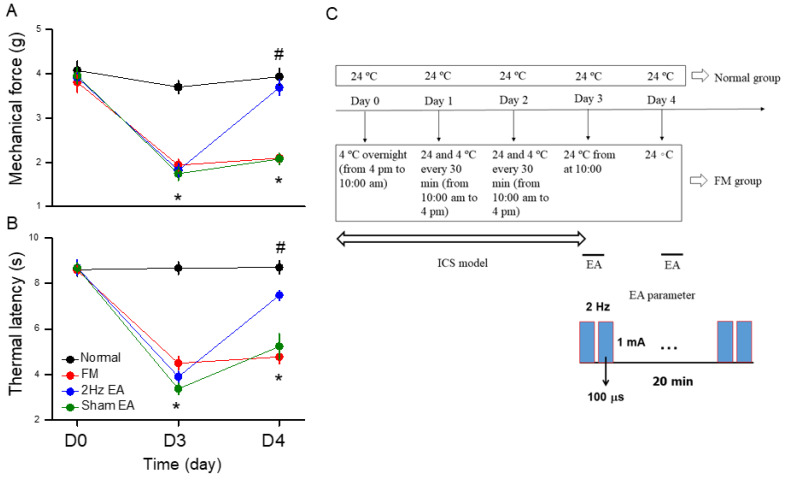
Mechanical and thermal hypersensitivity of mice in all groups. (**A**) The mechanical threshold obtained in the von Frey filament test; (**B**) thermal latency in the Hargreaves’ test. (**C**) Illustration of ICS modeling and EA treatment. * Significant difference in comparison to the normal group. ^#^ Significant difference from the FM group. *n* = 9 per group.

To understand why EA can attenuate FM pain in mice, we first evaluated nociception using the von Frey and Hargraves’ tests for hyperalgesia assessment. After submitting all mice to cold stress for three uninterrupted days, the nociceptive responses were assessed to confirm the successful induction of FM pain. All mice presented drastically reduced mechanical thresholds in the hind paw from baseline to post-cold stress. These lower thresholds persisted throughout the observation period (Figure 1A, red circle, day 4: 2.09 ± 0.09 g, * *p* < 0.05, *n* = 9). Low-frequency 2 Hz EA considerably improved paw withdrawal thresholds compared to FM (Figure 1A, blue circle, day 4: 3.69 ± 0.18 g, ^#^ *p* < 0.05, *n* = 9). In contrast, sham EA did not have any effects, suggesting acupoint specificity (Figure 1A, green circle, day 4: 2.08 ± 0.12 g, * *p* < 0.05, *n* = 9). Similarly, the Hargreaves’ test was applied to determine whether cold stress affected the thermal latency of FM mice (Figure 1B, red circle, day 4: 4.77 ± 0.29 g, * *p* < 0.05, *n* = 9). Furthermore, 2 Hz EA reduced thermal hyperalgesia by increasing the thermal latency (Figure 1B, blue circle, day 4: 7.47 ± 0.21 g, ^#^ *p* < 0.05, *n* = 9). Moreover, decreased thermal latency was detected in the sham EA group (Figure 1B, green circle, day 4: 5.23 ± 0.55 g, * *p* < 0.05, *n* = 9), indicating the critical role of acupoint specificity in the control of FM pain. Figure 1C illustrates the experimental protocol of a mouse FM model and EA parameters.

### 3.2. EA Increased CB1 Receptor Levels to Attenuate Signaling of FM Pain in the Thalamus

**Figure 2:**
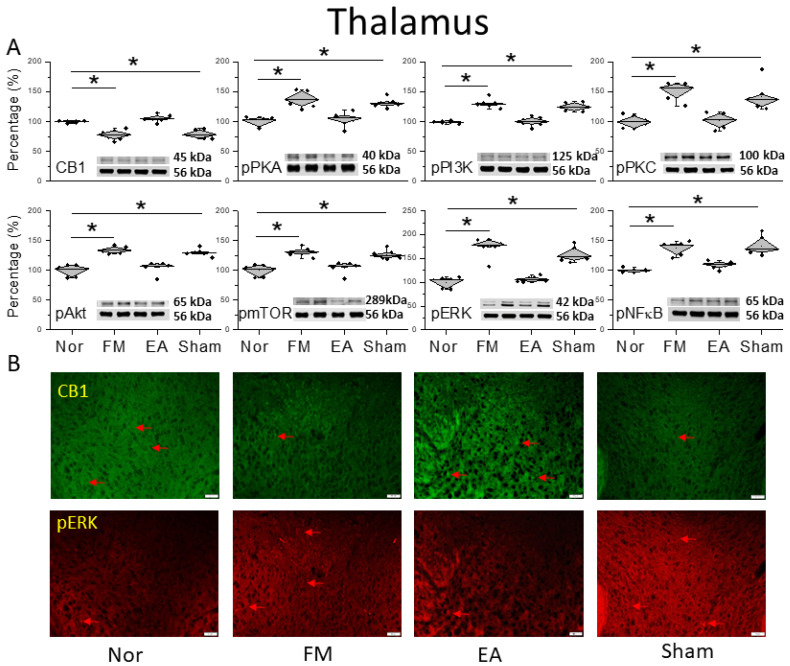
Protein levels of CB1 and related molecules in the thalamus. Western blots with four lanes indicating proteins in the following groups: normal, FM, FM + EA, FM + sham EA. (**A**) CB1, pPKA, pPI3K, pPKC, pAkt, pmTOR, pERK, and pNF-κB. * Significant difference between normal and others. *n* = 6. (**B**) CB1 and pERK immuno-positive (green or red) signals in the mice thalamus. Red arrows mean immune-positive signals. *n* = 3 in all groups.

Next, we wanted to determine how CB1 affects FM pain in the thalamus, so we performed a Western blot to quantify CB1 receptor levels. CB1 existed in the mice thalamus and the initiation of FM pain through cold stress reliably reduced CB1 expression (Figure 2A, * *p* < 0.05, *n* = 6). Low-frequency 2 Hz EA considerably increased CB1 expression after FM induction (Figure 2A, ^#^ *p* < 0.05, *n* = 6). Further, CB1 expression in the thalamus was further decreased after sham EA treatment (Figure 2A, * *p* < 0.05, *n* = 6). In addition, we evaluated that pain-related protein kinases or transcription factors were related to CB1 function, including phosphorylated protein kinase A (pPKA), phosphoinositide 3-kinase (pPI3K), and pPKC that were associated with pain signaling. There was a noticeably greater appearance of these kinases in the FM pain mice thalamus than normal mice (Figure 2A, * *p* < 0.05, *n* = 6), getting further decreased by 2 Hz EA treatment at ST36 (Figure 2A, ^#^ *p* < 0.05, *n* = 6). Such a result was not observed in the sham group, which indicated acupoint specificity (Figure 2A, * *p* < 0.05, *n* = 6). Similarly, pAkt and pmTOR levels were augmented after FM. A similar tendency was also observed in pERK and pNKκB expression levels in the thalamus of the FM group, which was related to the normal mice. Such increases were alleviated by EA but not by sham treatment (Figure 2A, * *p* < 0.05, *n* = 6). The qualitative confirmation in the thalamus was qualified via the immunofluorescence staining (Figure 2B, *n* = 3). The pictures of the thalamus presented significant attenuations of CB1 protein staining in the FM and FM + sham groups, in contrast with the control and FM + EA groups, which therefore had a significant increase in protein levels. In contrast, the reversal tendencies of pERK protein concentration were also found in the thalamus of mice (Figure 2B, *n* = 3).

### 3.3. Low-Frequency 2 Hz EA Reduced Cold FM Pain Through CB1 in the SSC

**Figure 3:**
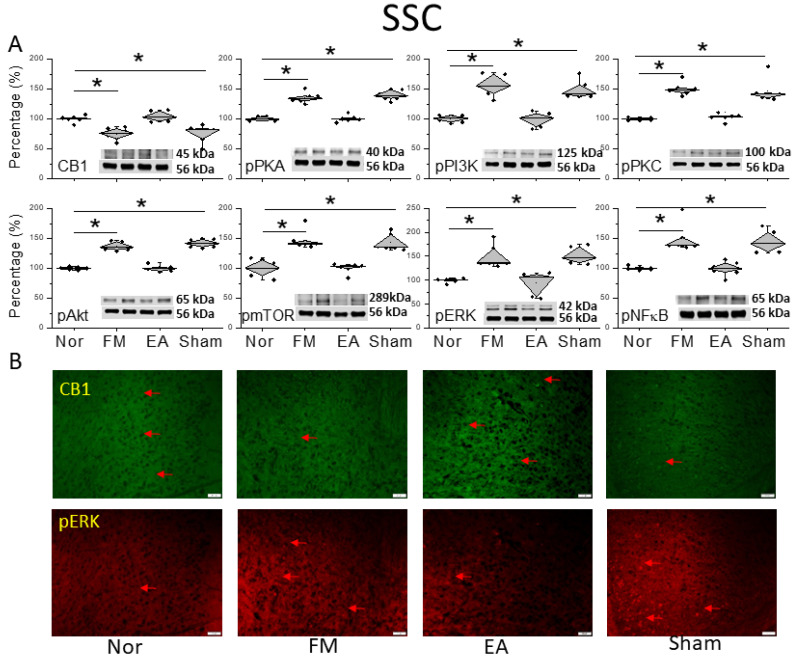
Protein levels of CB1 and related molecules in the SSC. Western blots with four lanes indicating proteins in the following groups: normal, FM, FM + EA, FM + sham EA. (**A**) CB1, pPKA, pPI3K, pPKC, pAkt, pmTOR, pERK, and pNF-κB protein levels. * Significant difference between normal and others. *n* = 6. (**B**) CB1 and pERK immuno-positive (green or red) signals in the mice SSC. Red arrows mean immune-positive signals. *n* = 3 in all groups.

To characterize the pain sensation of FM pain and EA in the CB1 relationship, we explored CB1 signaling in the SSC. Compared to the normal group, CB1 levels decreased 2 days after FM induction (Figure 3A, * *p* < 0.05, *n* = 6). This result indicates that CB1 attenuation caused FM pain in mice. This attenuation could be reversed by 2 Hz EA but not with sham treatment, suggesting a crucial effect of acupoint stimulation (Figure 3A, * *p* < 0.05, *n* = 6). We next detected nociceptive molecules in the SSC of FM and EA-treated animals. Compared with the normal group, the SSC in FM mice displayed increased pPKA, pPI3K, and pPKC levels (Figure 3A, * *p* < 0.05, *n* = 6), which were decreased by EA (Figure 3A, ^#^ *p* < 0.05, *n* = 6), but not by sham EA (Figure 3A, * *p* < 0.05, *n* = 6). Additionally, pAkt and pmTOR levels were increased in the SSC of FM mice. Comparable trends were observed in pERK and pNKκB expression (Figure 3A, * *p* < 0.05, *n* = 6). Immunofluorescence staining results showed a reduction in CB1 in the mice SSC after FM induction. The attenuation was then increased by 2 Hz EA treatment but not sham EA (Figure 3B, *n* = 3). The images from SSC indicated a significant augmentation of pERK expression in the FM and FM + sham groups but not the FM + EA group (Figure 3B, *n* = 3).

### 3.4. EA at ST36 Decreased Cold Stress-Induced FM Pain and Regulated TRPV1-CB1 Signaling in the ACC

**Figure 4:**
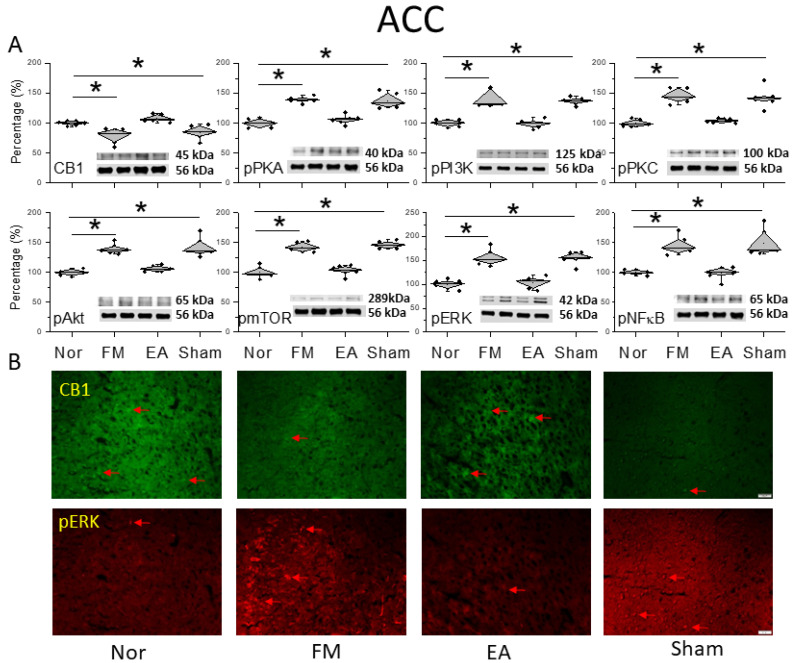
Protein levels of CB1 and related molecules in the ACC. Western blots with four lanes indicating proteins in the following groups: normal, FM, FM + EA, FM + sham EA. (**A**) CB1, pPKA, pPI3K, pPKC, pAkt, pmTOR, pERK, and pNF-κB protein levels. * Significant difference between normal and others. *n* = 6. (**B**) CB1 and pERK immuno-positive (green or red) signals in the mice ACC. Red arrows mean immune-positive signals. *n* = 3 in all groups.

Figure 4A shows CB1 expression before and after FM induction. The activity of the CB1 receptor significantly decreased in the ACC after FM (Figure 4A, * *p* < 0.05, *n* = 6), compared with normal mice. However, CB1 expression increased in the EA group, suggesting a crucial role of CB1 in FM ACC (Figure 4A, ^#^ *p* < 0.05, *n* = 6). This was not observed in the sham EA group (Figure 4A, * *p* < 0.05, *n* = 6). Similarly, pPKA, pPI3K, and pPKC were sequentially amplified after FM induction in mice (Figure 4A, * *p* < 0.05, *n* = 6), whereas the occurrences were improved by 2 Hz EA rather than sham EA mice (Figure 4A, * *p* < 0.05, *n* = 6). The formation of FM pain was also greater than before the expression of pAkt and pmTOR in the mice ACC (Figure 4A, * *p* < 0.05, *n* = 6), which is symptomatic of their important role in nociception formation. Additionally, 2 Hz EA but not sham EA attenuated their overexpression, as well as that of pERK and pNFκB (Figure 4A, * *p* < 0.05, *n* = 6). The Western blot quantitative results in ACC were additionally perceived through immunofluorescence depicted in Figure 4B. The pictures of the ACC revealed diverse trends of a reduced expression of CB1 and increased pERK protein staining in the FM and FM + sham groups but not in the FM + EA mice (Figure 4B, *n* = 3).

### 3.5. CB1 Regulation or Chemogenetic Modulation Affected Pain and Directly Targeted the CB1 Receptor in the SSC or ACC

**Figure 5:**
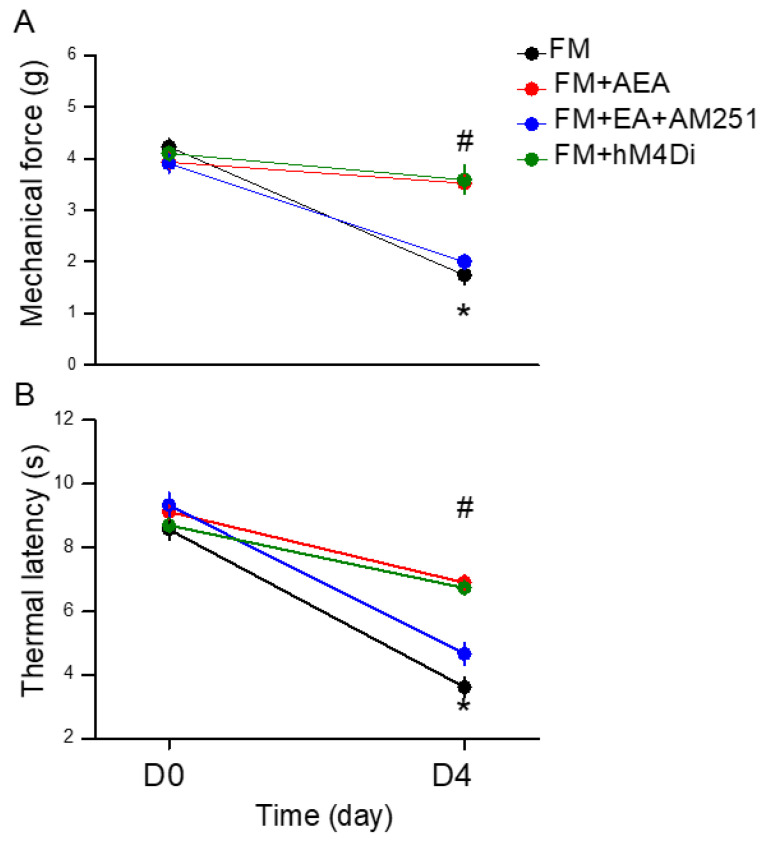
Mechanical and thermal hypersensitivity of mice in all groups: FM, FM + AEA, FM + EA + AM251, FM + hM4Di. (**A**) The mechanical threshold obtained in the von Frey filament test; (**B**) thermal latency in the Hargreaves’ test. * Significant difference in comparison to the normal group. ^#^ Significant difference from the FM group. *n* = 9 per group.

We further investigated if EA can modulate CB1 expression to relieve mice FM pain. To test whether CB1 activation could regulate mechanical hyperalgesia, we injected the CB1 agonist AEA into the intracerebral ventricle. The von Frey filament test indicated that AEA injection significantly attenuated mechanical hyperalgesia (Figure 5A, red circle, day 4: 3.53 ± 0.15 g, * *p* < 0.05, *n* = 9). We then used a CB1 receptor antagonist, AM251, to check if EA’s pain-relieving effects involved CB1. EA’s analgesic effect disappeared when AM251 was used (Figure 5A, blue circle, day 4: 2.01 ± 0.08 g, ^#^ *p* < 0.05, *n* = 9). We further used hM4Di DREADD to check if the inhibition of adenylyl cyclase (Gi) can alleviate FM-initiated mechanical hyperalgesia. The chemogenetic inhibition of SSC neurons, initiated by CNO injection, significantly diminished mechanical pain (Figure 5A, green circle, day 4: 3.59 ± 0.27 g, * *p* < 0.05, *n* = 9). Similar results were also observed in thermal latency (Figure 5B, * *p* < 0.05, *n* = 9).

**Figure 6:**
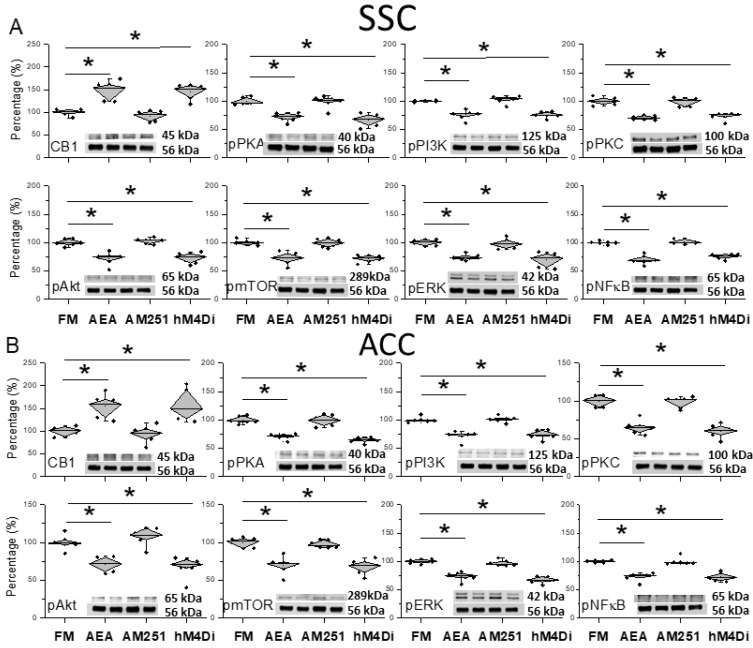
Protein levels of CB1 and related molecules in the SSC and ACC. Western blots with four lanes indicating proteins in the following groups: FM, FM + AEA, FM + EA + AM251, and FM + hM4Di. (**A**) CB1, pPKA, pPI3K, pPKC, pAkt, pmTOR, pERK, and pNF-kB in the SSC. (**B**) CB1, pPKA, pPI3K, pPKC, pAkt, pmTOR, pERK, and pNF-kB in the ACC. * Significant difference between normal and others. *n* = 6.

We further examined if modulating the CB1 receptor can modulate CB1 and related kinases in the mice FM model. ICS significantly decreased CB1 expression in the SSC. Injecting AEA into the SSC significantly increased CB1 levels in the SSC (Figure 6A, * *p* < 0.05, *n* = 6). In contrast, AEA injection significantly attenuated pPKA, pPI3K, and pPKC overexpression (Figure 6A, * *p* < 0.05, *n* = 6). Similar results were observed for pAkt, pmTOR, pERK, and pNKκB (Figure 6A, * *p* < 0.05, *n* = 6). Using AM251 injection concomitantly to EA, we observed that CB1 levels were similar to those of the FM model, suggesting that the main effect of EA was via the activation of the CB1 receptor (Figure 6A, ^#^ *p* < 0.05, *n* = 6). Finally, we precisely inhibited SSC layer 2/3 neurons by chemogenetic inhibition, which significantly increased CB1 levels. Simultaneously, the inhibition of SSC layer 2/3 neurons reduced the overexpression of pain-related kinases (Figure 6A, * *p* < 0.05, *n* = 6).

Similar data were obtained in the ACC, suggesting a downstream neuronal circuit from SSC. Our results showed increased CB1 expression after AEA injection (Figure 6B, * *p* < 0.05, *n* = 6). EA cannot increase the protein levels of CB1 due to AM251 administration (Figure 6B, ^#^ *p* < 0.05, *n* = 6). Further, chemogenetic inhibition significantly increased the CB1 protein contained in the ACC (Figure 6B, * *p* < 0.05, *n* = 6). In contrast, pPKA, pPI3K, and pPKC levels all decreased after AEA injection, suggesting a crucial role of CB1 in this model (Figure 6B, * *p* < 0.05, *n* = 6). Diverse results were obtained in EA-treated mice with AM251 administration (Figure 6B, ^#^ *p* < 0.05, *n* = 6). Furthermore, the chemogenetic inhibition of SSC reversed the effect of EA on pain-related kinases (Figure 6B, * *p* < 0.05, *n* = 6). Similar trends were obtained for pAkt, pmTOR, pERK, and pNFκB (Figure 6B, * *p* < 0.05, *n* = 6).

## 4. Discussion

Here, we performed ICS to develop a mouse FM model accompanied by mechanical and thermal hyperalgesia, similar to symptoms in clinical FM patients. These phenotypes were confirmed by von Frey and Hargraves’ test. EA but not sham EA significantly attenuated hyperalgesia. Furthermore, a Western blot and immunofluorescence analysis showed decreased CB1 and increased nociceptive kinase levels. Moreover, EA but not sham EA dramatically reversed these effects.

A recent article mentioned that the myofascial technique approach was beneficial in treating fibromyalgia via reducing pain, central sensitization, and negative emotional symptoms, and improving sleep quality [35]. Martínez-Pozas et al. indicated a beneficial effect of orthopedic manual therapy in mechanical hyperalgesia in chronic musculoskeletal pain patients [36]. Previous research reported CB expression in the thalamus, a crucial region for pain transduction, of a rat model of chronic neuropathic pain characterized by thermal and tactile hyperalgesia. In that study, the thalamic CB triggered antinociceptive effects. In addition, cannabinoids could effectively prevent thermal and tactile hyperalgesia in a tibial nerve injury model [37]. Recent reports suggest that abnormal activities in the zona incerta and posterior complex of the thalamus are involved in neuropathic pain. However, the specific cellular mechanisms and cell types involved in this nociception circuit remain unclear. Wang et al. indicated that parvalbumin-positive neuronal projections from the zona incerta to the posterior complex of the thalamus are crucial for painful behaviors. In addition, the inhibition of neuronal circuit activity reduces nociceptive responses. Similarly, CB1 is expressed in axon terminals of neurons in this circuit, and cannabinoid administration significantly attenuates nociceptive responses via presynaptic inhibition [38]. The thalamic reticular nucleus (TRN) is reportedly involved in sensation, attention, and sleep. Ding et al. considered that the connections from the TRN to the ventroposterior thalamus are crucial in chronic sleep disruption (CSD)-induced hyperalgesia in mice. Further, CB1 activity in the TRN decreased after CSD, suggesting an important role in pain development [39]. In this study, we demonstrated lower CB1 levels in our mouse FM model, which could be reversed by 2 Hz EA but not sham EA.

Anandamide is a known endogenous ligand of CB. Bir and Ercan identified that injecting anandamide intrathecally decreased the conduction of somatosensory-evoked potentials. Cannabinoids are effective modulators of neuronal circuit activity in the rodent brain. CB1 activation significantly attenuates sensory-evoked responses by diminishing excitatory neurotransmission [40]. Further, in the neocortex, CB1 receptor levels were high in layers 2/3 and 5 of the somatosensory cortex. They also verified that cannabinoid signaling in layer 2/3 of the somatosensory cortex is geared to both g-aminobutyric acid (GABA) and glutamate neurotransmission. Cannabinoids have several properties in either excitation or inhibition with somatosensory cortical layers. Moreover, cannabinoidal signaling in layer 5 was suggested to decrease the efficacy of excitatory signals [41]. In somatosensory cortex layer 2/3, cannabinoidal signaling can inhibit GABA release and then cause an enhanced excitability of pyramidal neurons. Hsieh and Levine reported that CB1 activation reliably augmented backpropagating action potential-induced calcium signals in the apical dendrites of layer 2/3 of the somatosensory cortex. This is an effect reversed by a GABA_A_ receptor antagonist [42]. Moreover, brain-derived neurotrophic factor (BDNF) increased excitatory neurotransmission by increasing presynaptic glutamate release. In addition, the outcome of BDNF in excitatory transmission in SSC was uncovered by blocking CB1 receptors [43].

Cannabidiol is a phytocannabinoid from *Cannabis sativa* used to treat several pain conditions. Genaro et al. verified the effects of cannabidiol, which was injected both systemically or locally into ACC regions for postoperative pain and spontaneous incision pain. They suggest that cannabidiol has an analgesic effect both in the local ACC region as well as systemic administration [44]. Improving understanding of brain neuronal circuits and mechanisms in modulating endogenous antinociception is essential for a fundamental perspective and to further develop the identification of novel therapeutic approaches for pain relief. ACC is involved in the emotional component of pain sensation. In fact, Corcoran et al. showed that CB2 receptors play a main role in stress-induced and fear-conditioned analgesia within the ACC in rats [45]. Further, the intra-ACC administration of a bioactive lipid diminished the first and second phases of formalin pain, effects mitigated by AM251, a CB1 antagonist [46]. Recently, the hyperfunction of pyramidal neurons in the ACC in chronic inflammatory pain was related to the malfunction of CB. Similarly, EA significantly decreased the hyperfunction of pyramidal neurons via increased endocannabinoid and CB1 receptor levels. In addition, CB1 receptors in the ACC could reduce the N-methyl-D-aspartate receptor (NMDA) NR1 subunits via histidine triad nucleotide-binding protein 1 (HINT1) in the ACC [47]. During inflammation, the hypothalamus, amygdala, hippocampus, and brain cortex were influenced and induced hyperfunction [48,49]. The chemogenetic technique was often used to modulate brain functions especially in pain management. Yi et al. reported that the chemogenetic modulation of microglia attenuated neuroinflammation and neuropathic pain in mice. They indicated that microglial Gi DREADD activation inhibited spinal nerve transection-induced microglial activity as well as chronic pain [50]. Perez-Sanchez et al. also initiated a humanized chemogenetic method to diminish pain behavior in human sensory neurons [51]. Moreover, Cui et al. determined that the chemogenetic inhibition of anterior paraventricular thalamic nucleus glutamatergic neurons significantly reduced mechanical pain in inflamed mice [52].

## 5. Conclusions

The present results imply that mice suffering from ICS had a significantly lower mechanical threshold and thermal latency as well as lower CB1 levels. The decrease in CB1 levels was reversed by 2 Hz EA but not sham EA. Serial pain-related protein kinases were augmented by ICS modeling and relieved by 2 Hz EA. Our innovative discoveries suggest a role for CB1 signaling in the communication between EA and FM, suggesting its potential as a treatment target.

The main limitation of this research was that the CB1 signaling pathway was only shown in this ICS-initiated FM model. Post mortem clinical brain tissue of FM patients is required to confirm our findings. We also cannot ignore any other receptors in this FM model. In the near future, we need to explore the clinical application of CB1 as a treatment target for FM.

## Data Availability

The datasets supporting the conclusions of this article are included within the article.
